# Prostate Cancer

**DOI:** 10.1100/tsw.2011.79

**Published:** 2011-04-05

**Authors:** Scott Eggener

**Affiliations:** Urologic Oncology, University of Chicago Medical Center, Chicago, Illinois, USA

**Keywords:** prostate, cancer, surgery, PSA, screening, surveillance, database.

## Abstract

Prostate cancer continues to be a significant public health issue worldwide, particularly in countries where men have life expectancies long enough to clinically manifest the disease. In many countries, it remains one of the leading causes of cancer-related morbidity and mortality.

Although significant progress has been made over the past few decades, many elements regarding the diagnosis and management of patients with prostate cancer remain enigmatic. In this Prostate Cancer special issue, our expert authors present and examine clinically relevant topics and look ahead to future research.

Prostate cancer continues to be a significant public health issue worldwide, particularly in countries where men have life expectancies long enough to clinically manifest the disease. In many countries, it remains one of the leading causes of cancer-related morbidity and mortality. Although significant progress has been made over the past few decades (e.g., introduction of prostate specific antigen [PSA], refined radiotherapy and surgical techniques, novel systemic agents), many elements regarding the diagnosis and management of patients with prostate cancer remain enigmatic. For instance, significant controversy exists regarding the merits and limitations of PSA-based screening, namely who should be screened, how, when to stop, and when to proceed with biopsy. Upon diagnosis of clinically localized prostate cancer, a man is faced with many management options without definitive evidence favoring a uniform strategy.

To provide a meaningful overview of the various clinical care options, the talented authors in this special issue of Prostate Cancer for *TheScientificWorldJOURNAL: TSW Urology* have compiled the most contemporary and relevant evidence.

Since the introduction of PSA-based prostate cancer screening, the age-adjusted mortality rate from prostate cancer has decreased approximately 40% in the U.S. Along with the impressive number of men benefiting, estimates suggest that up to 23–56% of men are unnecessarily diagnosed ("overdiagnosis") or treated ("overtreatment"), essentially informed, and typically treated, of a disease that is highly unlikely to become clinically evident during their natural lifespan. Therefore, implicit in any screening effort is to identify individuals most likely to develop the disease, and most likely to benefit from prompt diagnosis and treatment. Ercole and Parekh[1] provide an overview of the risk factors for developing prostate cancer and tools available to predict that risk. The authors also explore data from multiple, randomized, chemoprevention trials as potential methods of minimizing the individual and societal burdens of prostate cancer.

PSA remains the primary component of prostate cancer screening and, in my mind, has been definitively shown to save lives. Depending on which patients are screened and how they are screened, the number of men required to receive a PSA to prevent one death ranges from 700 to 1400, and the number of men required to have their prostate cancer treated to prevent one death ranges from 5 to 48. Based on its imperfect operating characteristics, PSA can lead to the diagnosis of men with indolent cancers with an exceedingly low likelihood of progression. Alternatively, PSA-based efforts may not detect potentially lethal cancers, as they either do not produce PSA or are locally advanced/metastatic at diagnosis. Prostate cancer screening has been an iterative process with introduction of PSA isoforms (e.g., free, complexed), variable PSA-based calculations (e.g., density, velocity), and novel urine and serum biomarkers. Tosoian and Loeb[2] commendably review the landscape of screening techniques and the search for a more perfect screening test.

When a man is diagnosed with clinically localized prostate cancer, presuming he has an estimated life expectancy of at least 7–10 years, there are multiple management options at his disposal. Approximately 95% of these men elect active surveillance, radiation therapy, or surgery. With a dearth of prospectively collected, mature, randomized studies comparing these options, this decision can be understandably challenging for both the individual and his physician. We are fortunate to have detailed overviews of the risks, benefits, and rationale of these three options. First, Wu and Dall'Era[3] review active surveillance, a method of carefully selecting patients with low-risk cancer characteristics, closely observing patient and cancer factors, and potentially treating the cancer at a later date, if deemed necessary. Second, Lowrance, Tarin, and Shariat[4] discuss the two most common surgical techniques, robotic radical prostatectomy and open retropubic radical prostatectomy, with special emphasis on comparing the best available nonrandomized data to inform decision making. Lastly, Choe and Liauw[5] share the multitude of radiation therapy techniques, indications, and randomized trial data.

With a dearth of randomized trials comparing treatment strategies for men with localized prostate cancer, large databases can be explored to examine treatment patterns and outcomes. While inherently embedded with methodologic limitations, they nevertheless provide useful metrics, when interpreted appropriately. Su and Jang[6] commendably provide an overview of the available and commonly used large databases harnessed to explore clinically relevant questions.

Our authors have collectively and expertly provided our Prostate Cancer Special Issue with clinically relevant topics that we hope you will find useful and informative as you page through them.
